# Prospero-related homeobox 1 (Prox1) at the crossroads of diverse pathways during adult neural fate specification

**DOI:** 10.3389/fncel.2014.00454

**Published:** 2015-01-26

**Authors:** Athanasios Stergiopoulos, Maximilianos Elkouris, Panagiotis K. Politis

**Affiliations:** Center for Basic Research, Biomedical Research Foundation of the Academy of AthensAthens, Greece

**Keywords:** Prox1, adult neurogenesis, dentate gyrus, hippocampus, neural differentiation, neuronal progenitors, nuclear receptors

## Abstract

Over the last decades, adult neurogenesis in the central nervous system (CNS) has emerged as a fundamental process underlying physiology and disease. Recent evidence indicates that the homeobox transcription factor Prox1 is a critical intrinsic regulator of neurogenesis in the embryonic CNS and adult dentate gyrus (DG) of the hippocampus, acting in multiple ways and instructed by extrinsic cues and intrinsic factors. In the embryonic CNS, Prox1 is mechanistically involved in the regulation of proliferation vs. differentiation decisions of neural stem cells (NSCs), promoting cell cycle exit and neuronal differentiation, while inhibiting astrogliogenesis. During the complex differentiation events in adult hippocampal neurogenesis, Prox1 is required for maintenance of intermediate progenitors (IPs), differentiation and maturation of glutamatergic interneurons, as well as specification of DG cell identity over CA3 pyramidal fate. The mechanism by which Prox1 exerts multiple functions involves distinct signaling pathways currently not fully highlighted. In this mini-review, we thoroughly discuss the Prox1-dependent phenotypes and molecular pathways in adult neurogenesis in relation to different upstream signaling cues and cell fate determinants. In addition, we discuss the possibility that Prox1 may act as a cross-talk point between diverse signaling cascades to achieve specific outcomes during adult neurogenesis.

## Introduction

It is now firmly established that active neurogenesis continues throughout life in discrete regions of the central nervous system (CNS) of all mammals, including humans (Eriksson et al., 1998; Temple and Alvarez-Buylla, 1999; Gage, 2000). This revolutionizing finding unraveled the pivotal role of neural stem cells (NSCs) in the adult brain and generated new hope for the treatment of brain impairment during aging and neurodegenerative disorders. Adult neurogenesis is particularly prominent in the subgranular zone (SGZ) of the dentate gyrus (DG) in the hippocampus (Altman and Das, 1965; Seri et al., 2001) and the subventricular zone (SVZ) of the lateral ventricles (Garcia-Verdugo et al., 1998; Johansson et al., 1999). In the hippocampus, an area associated with learning and memory, neurogenesis may play a role in enhancing learning ability, cognitive performance and facilitating the formation of new memories (Deng et al., 2010). The formation of the DG is a complex process that involves cell proliferation, migration and neuronal differentiation (Pleasure et al., 2000). In the SGZ, adult NSCs generate intermediate progenitors (IPs), that eventually differentiate into excitatory glutamatergic granule neurons (Seri et al., 2001). In particular, stem cells with radial processes (radial glia-like cells, type-1 cells) give rise to IPs with high proliferative activity (type-2 cells). A subset of these cells still expresses glial markers, but lack radial processes (type-2a). Another subset, type-2b cells are originating from type-1 cells as well and show characteristics of neuronal lineage, expressing transcription factors such as Prox1 and NeuroD1 (Steiner et al., 2006). Type-2 cells respond to physiological stimuli such as physical exercise (Kronenberg et al., 2003) or pharmacological stimulation (Encinas et al., 2006), and are prone to differentiate into neuronal committed neuroblasts (type-3 cells). Under normal conditions, type-3 cells exert little proliferative activity, whereas under experimental seizures mimicking pathological conditions, type-3 cells show increased proliferation (Jessberger et al., 2005). Once they exit cell cycle, newly formed neurons send their axons to target areas such as the CA2 and CA3 of hippocampus, where they form appropriate synapses. The balanced coordination of these processes is essential for tissue homeostasis in the adult brain.

**Figure 1:**
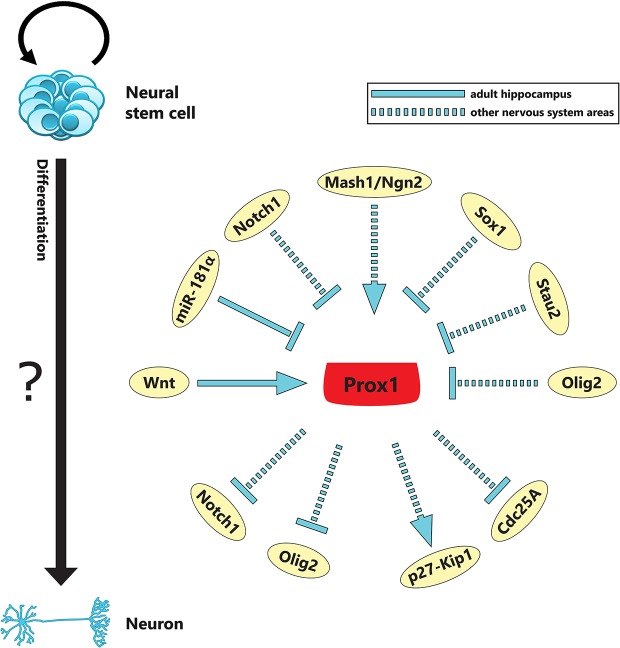
**Schematic depiction of the involvement of Prox1 in diverse critical pathways that regulate neurogenesis during adult and embryonic NSC fate specification**. Prox1 may act as a key cross-talk point between upstream and downstream signaling processes to achieve specific outcomes during neurogenesis in the adult DG of the hippocampus (i.e., canonical Wnt, *miR-181α*; *continuous line*) and the embryonic CNS (i.e., Notch1, *Mash1/Ngn2* proneural genes, Sox1, Stau2, Olig2; *discontinuous line*). Furthermore, *Prox1* acts as tumor suppressor gene in neuroblastoma cells by regulating basic components of the cell cycle machinery (i.e., p27-Kip1, Cdc25A) (see also Table 1).

Prox1, a homeobox transcription factor, has been suggested to play key roles in adult neurogenesis of the hippocampus. Interestingly, Prospero, the *Drosophila* homologue of Prox1 in vertebrates, is a critical regulator of the balance between self-renewal and differentiation in NSCs (Li and Vaessin, 2000; Choksi et al., 2006). Prospero suppresses the genetic program for self-renewal of NSCs and cell cycle progression, while it activates genes necessary for terminal neuronal differentiation (Choksi et al., 2006; Southall and Brand, 2009). Neuroblasts that lack Prospero form tumors in the embryonic nervous system of *Drosophila* (Choksi et al., 2006). In vertebrates, Prox1 is a key regulator for the generation of many organs during embryogenesis such as the brain, spinal cord, retina, lens, liver, pancreas and endothelial lymphatic system (Oliver et al., 1993; Tomarev et al., 1996; Wigle and Oliver, 1999; Wigle et al., 1999; Sosa-Pineda et al., 2000; Dyer et al., 2003; Wang et al., 2005; Lavado and Oliver, 2007; Misra et al., 2008; Kaltezioti et al., 2010). *Prox1* knock out embryos die before birth due to multiple developmental defects (Wigle and Oliver, 1999; Wigle et al., 1999). Although the role of Prox1 in the development of lymphatic vasculature, liver, pancreas, heart and lens has received much attention in previous studies (Wigle et al., 1999; Sosa-Pineda et al., 2000; Burke and Oliver, 2002; Risebro et al., 2009), its potential role in neurogenesis has just begun to emerge (Wigle and Oliver, 1999; Wigle et al., 2002; Lavado and Oliver, 2007; Misra et al., 2008; Kaltezioti et al., 2010, 2014; Lavado et al., 2010). Accordingly, we have recently unraveled the key role of Prox1 in regulating the fine balance between proliferation and differentiation of NSCs during spinal cord development and neuroblastoma cancer progression (Kaltezioti et al., 2010; Foskolou et al., 2013). In particular, we showed that Prox1 promotes neurogenesis and inhibits astrogliogenesis and self-renewal of embryonic NSCs. We also reported that Prox1 suppresses cell cycle progression and proliferation of neuroblastoma cancer cells via a direct action in basic components of the cell cycle machinery (Foskolou et al., 2013). Moreover, we very recently showed that Prox1 controls binary fate decisions between motor neurons and V2 interneurons in the developing spinal cord via direct repression of *Olig2* gene expression (Kaltezioti et al., 2014). Collectively, these observations indicate a central role for Prox1 in neural development (Figure 1; Table 1).

**Table 1:** **List of Prox1-dependent phenotypes and molecular pathways implicated in adult and embryonic neural fate specification (see also Figure 1)**.

Nervous tissue	Prox1 Role/Regulation	Organism	References
Dentate Gyrus (DG) (embryonic & adult hippocampus)	Maturation of granule neurons; NSC proliferation and maintenance; survival of intermediate progenitors (IPs)	Mouse	Lavado and Oliver (2007), Lavado et al. (2010), Karalay and Jessberger (2011), Karalay et al. (2011)
Adult Dentate Gyrus	Canonical Wnt signaling directly regulates Prox1 expression; Prox1 induces neuronal differentiation	Mouse	Karalay and Jessberger (2011), Karalay et al. (2011)
Postnatal & Adult Hippocampus	Postmitotic specification of DG granule cell identity over CA3 pyramidal cell fate	Mouse	Iwano et al. (2012)
Adult Hippocampus	*miR-181α* overexpression mimics reduced Prox1 levels and increased Notch1 levels; induction of astrocytic differentiation	Mouse	Xu et al. (2014)
Adult Hippocampus	Robust promotion of DG cell replacement (transplantation studies)	Rat	Chen et al. (2011)
Embryonic Spinal Cord	Regulation of Notch1-Mediated Inhibition of Neurogenesis; induction of neurogenesis; inhibition of astrogliogenesis and self-renewal of NSCs	Mouse Chicken	Kaltezioti et al. (2010)
Embryonic Spinal Cord	Liver receptor homologue-1 (LRH-1/NR5A2) facilitates the Prox1-mediated inhibition of Notch1 signaling	Mouse Chicken	Kaltezioti et al. (2010), Stergiopoulos and Politis (2013)
Embryonic Spinal Cord	Regulation of binary fate decisions between motor neurons and V2 interneurons via direct repression of *Olig2* gene expression	Mouse Chicken	Kaltezioti et al. (2014)
Embryonic Central Nervous System (CNS)	Mash1 and Ngn2 induce Prox1 expression; reduced Prox1 levels in *Mash1−/–* mice	Mouse Chicken	Misra et al. (2008), Torii et al. (1999)
Embryonic Subventricular Zone (SVZ)	Sox1 maintains the pool of cortical progenitors by suppressing Prox1-induced neurogenesis	Mouse	Elkouris et al. (2011)
Embryonic Cortex	Staufen2 (Stau2)-dependent RNA complex represses *Prox1* mRNA; reduced neurogenesis	Mouse	Vessey et al. (2012)
Nervous System- related Cancers	Tumor suppressor gene by regulating Cyclins, p27-Kip1 and Cdc25A; induction of cell cycle arrest	Mouse Human	Foskolou et al. (2013)
*Drosophila* Nervous System (prospero)	Inhibition of the genetic program for NSC self-renewal & cell cycle progression; *Prox1−/–* neuroblasts form tumors (embryonic nervous system); Activation of genes necessary for terminal neuronal differentiation	Drosophila melanogaster	Li and Vaessin (2000), Choksi et al. (2006), Southall and Brand (2009)

## Prox1 is a key player in hippocampal neurogenesis

In the brain, Prox1 is detected in various regions, including cortex (CTX), DG, thalamus, hypothalamus and cerebellum during prenatal and postnatal developmental stages and adulthood (Oliver et al., 1993; Galeeva et al., 2007; Lavado and Oliver, 2007). Prox1 has been proposed to act as a master regulator of hippocampal neurogenesis (Lavado et al., 2010; Karalay and Jessberger, 2011; Karalay et al., 2011; Iwano et al., 2012; Sugiyama et al., 2014). During early stages of hippocampal formation, the DG is generated from NSCs of the dentate neuroepithelium (DNE), a region highly expressing Prox1 (Lavado and Oliver, 2007). At later prenatal and postnatal developmental stages, Prox1 is detected in type-2 IPs (Dcx+, Tbr2+ cells) that reside in the SGZ, in type-2 IPs and early born neurons (NeuroD+ cells) along the dentate migratory stream (DMS) and finally in the mature granule cells (calbindin+) that mainly compose the adult DG (Lavado et al., 2010; Iwano et al., 2012; Sugiyama et al., 2014). Therefore, it is commonly used as a specific marker for the DG cell lineage (Oliver et al., 1993; Galeeva et al., 2007; Lavado and Oliver, 2007). Most importantly, recent *in vivo* data support the notion that Prox1 is necessary for IP maintenance and survival as well as granule neuron differentiation and maturation in embryonic and adult hippocampus (Lavado et al., 2010; Karalay and Jessberger, 2011; Karalay et al., 2011; Iwano et al., 2012; Table 1). Interestingly, Prox1-expressing IPs are required for adult NSC self-maintenance in the DG through a non-cell autonomous regulatory feedback mechanism (Lavado et al., 2010). Moreover, Prox1 postmitotically specifies DG granule cell identity over CA3 pyramidal cell fate in the early postnatal and adult hippocampus (Lavado and Oliver, 2007; Iwano et al., 2012). In addition, transplantation studies have shown that only donor cells expressing Prox1 can promote robust DG cell replacement in the adult rat hippocampus highlighting the critical role of Prox1 in adult DG neurogenesis (Chen et al., 2011).

Apparently, all Prox1 functions in the developing and adult hippocampus cannot be explained by one control mechanism. The temporal and spatial expression of Prox1 suggests a complex regulatory mode of action in a cell-context manner. The mechanism by which Prox1 exerts multiple functions involves distinct signaling pathways currently not fully understood. In the adult DG, Prox1-expressing precursor cells respond to several stimuli, such as growth factors (Lee and Agoston, 2010), physical activity, enriched environments and kainic-acid induced seizures, which contribute to neuronal regeneration and plasticity (Steiner et al., 2008). Most importantly, Prox1 is sufficient to direct the differentiation of progenitor cells into mature neurons *in vivo*. Niche-derived signals that determine cell fate and ensure life-long neurogenesis in the adult mammalian DG are recently linked to Prox1. In particular, canonical Wnt signaling emanated by hippocampal astrocytes has been shown to promote ectopic Prox1 activation by direct binding of β-catenin on the Prox1 TCF/LEF enhancer sites, hence triggering Prox1-mediated neuronal differentiation (Karalay and Jessberger, 2011; Karalay et al., 2011). Furthermore, recent evidence highlighted the important role of microRNAs in the regulation of Prox1 activity. In particular, *miR-181a* has been shown to directly bind to the 3’UTR Prox1 sequence while its overexpression mimics reduced Prox1 levels, and ultimately drives adult hippocampal progenitors into an astrocytic fate (Kazenwadel et al., 2010; Xu et al., 2014; Figure 1). However, the cell-autonomous transcription program that is activated by Prox1 in adult NSCs to instructively direct neuronal differentiation is still elusive.

## Insights for Prox1 molecular function in the adult hippocampus from other areas of the nervous system

The identification of Prox1 downstream targets that control NSC maintenance and differentiation is of cardinal importance in order to delineate the stage and time specific role of Prox1 in adult hippocampal neurogenesis. Towards this aim, functional evidence suggests that Prox1 counteracts Notch1 signaling via direct suppression of *Notch1* gene expression to promote neurogenesis and inhibit astrogliogenesis and self-renewal of NSCs in the developing spinal cord (Kaltezioti et al., 2010). Considering the important role of Notch1 in promoting maintenance of NSCs during adult-SGZ neurogenesis (Ables et al., 2010; Ehm et al., 2010; Lugert et al., 2010), we could hypothesize that Prox1 may affect adult neuronal progenitors by directly regulating Notch1 signaling. In adult hippocampal neural progenitor cells, decreased Prox1 levels by *miR-181a* overexpression was accompanied by increased Notch1 levels further providing evidence for the mechanistic association of these factors (Xu et al., 2014). Moreover, we recently showed that *Prox1* acts as tumor suppressor gene in nervous system related cancers by regulating basic components of the cell cycle machinery, including Cyclins, p27-Kip1 and Cdc25A, to induce cell cycle arrest (Foskolou et al., 2013). This anti-proliferative action of Prox1 could also be involved in the exit of adult NSCs from the cell cycle and induction of terminal neuronal differentiation of the adult-SGZ-derived neurons (Figure 1; Table 1).

Evidence from the embryonic brain on Prox1 regulation and activity might as well provide useful information towards the discovery of novel cellular and molecular mechanisms involved in adult hippocampal neurogenesis. Critical pathways that maintain the balance between NSC maintenance and differentiation, including Notch, Sox and proneural factors, have been linked to Prox1 activity (Torii et al., 1999; Misra et al., 2008; Kaltezioti et al., 2010; Elkouris et al., 2011). The SoxB1 subfamily (Sox1-3) is expressed by NSCs and IPs in the developing nervous system, where these factors maintain these cells in an undifferentiated state while suppressing neuronal differentiation (Remboutsika et al., 2011; Mandalos et al., 2012, 2014; Karnavas et al., 2013). One mechanism is mediated by Sox1 that maintains the pool of cortical progenitor cells by suppressing Prox1-induced neurogenesis in the mammalian embryonic SVZ (Elkouris et al., 2011). All SoxB1 transcription factors (Sox1-3) mark both radial astrocytes (type 1 cells) and early progenitor cells (type 2a cells) within the adult DG providing evidence for their potential implication in Prox1 regulation (Steiner et al., 2006; Wang et al., 2006; Venere et al., 2012). Sox21, another member of the *SoxB* genes, is also a critical regulator of adult neurogenesis in mouse hippocampus. Loss of Sox21 impairs transition of progenitor cells from type 2a to type 2b, thereby reducing subsequent production of new neurons in the adult DG (Matsuda et al., 2012). Mechanistically, Sox21 represses expression of the Notch-responsive gene *Hes5* at the transcriptional level (Matsuda et al., 2012). Prox1 may possibly play a major role at the point where the Notch and Sox pathways intersect to control neurogenesis in the adult hippocampus.

In addition, Prox1 in the embryonic CNS is induced by proneural genes and is required for implementation of their neurogenic program (Torii et al., 1999; Misra et al., 2008). In particular, Prox1 is partially co-expressed with Mash1 and Ngn2 in the SVZ of murine brain and chick spinal cord during the initial stages of neurogenesis. Overexpression of these factors is sufficient to induce Prox1 expression (Torii et al., 1999; Misra et al., 2008). Conversely, Prox1 levels are reduced in the embryonic brain of *Mash1* knockout mice (Torii et al., 1999), further suggesting that Prox1 expression during neurogenesis is dependent on proneural genes. The epistatic relationship between proneural genes and Prox1 may also be in action during adult neurogenesis and participate in mediating the important roles of these genes in hippocampal neurogenesis. Recently, another level of complexity in Prox1 activity throughout the embryonic brain has been added by RNA binding proteins that control *Prox1* mRNA stability. Staufen2 (Stau2)-dependent RNA complex is essential for appropriate precursor cell maintenance in embryonic cortical progenitor cells by binding and repressing *Prox1* mRNA (Vessey et al., 2012). In this study, genetic knockdown of *Stau2* causes enhanced expression of *Prox1* mRNA and subsequently leads to inappropriate neurogenesis, which could be potentially related to adult hippocampal neurogenesis (Figure 1; Table 1).

## Lessons on Prox1 function from other non-neural tissues

In other tissues, additional signaling factors, including HIF-1α/HIF-2α, LSD1 and Nuclear receptors (COUP-TFII, LRH-1, RORs), directly or indirectly affect Prox1 expression or activity (Table 2). Many of these factors are also key players in neural cell fate decisions raising the intriguing possibility that these factors may contribute to Prox1 mode of action in neurogenesis during development and adulthood. As an example, during lymphatic development, COUP-TFII (chicken ovalbumin upstream promoter–transcription factor II/NR2F2), a transcription factor also related to neuronal development, specifies lymphatic endothelial identity by physically and functionally interacting with Prox1 and specifically by forming heterodimers with Prox1 thereby maintaining the expression of *FGFR-3* and *VEGFR-3* genes and leading to the repression of the Notch target genes *Hey1/2* (Lee et al., 2009; Yamazaki et al., 2009; Aranguren et al., 2013). Moreover, COUP-TFII is necessary for the initiation and early maintenance of Prox1 expression during specification and differentiation of lymphatic endothelial cells (Srinivasan et al., 2010). Interestingly, apart from being expressed in Prox1 positive cells in the ganglionic eminences and in migrating cortical interneurons during forebrain development (Kanatani et al., 2008; Lin et al., 2011; Cai et al., 2013; Rubin and Kessaris, 2013), COUP-TFII is also detected in restricted populations of glutamatergic pyramidal cells and GABAergic neurons in the adult rat hippocampus (Fuentealba et al., 2010). This cell type-specific role of COUP-TFII could suggest potential correlation and/or interaction with Prox1 in cell fate decisions and neuronal maturation during adult hippocampal neurogenesis.

**Table 2:** Selected list of Prox1 regulatory roles in non-neural cells^*^.

Non-nervous tissues	Prox1 Role/Regulation	Organism	References
Enterohepatic System	Co-repressor partner for LRH-1; overlapping expression patterns; Prox1 and LRH-1 coordinately regulate the characteristics of hepatocytes	Mouse Human	Qin et al. (2004), Steffensen et al. (2004), Kamiya et al. (2008), Qin et al. (2009), Stein et al. (2014)
Hepatocytes	Interaction with LSD1 (lysine-specific demethylase 1) and recruitment of the repressive LSD1/NuRD complex to specific loci; co-repression of transcription via epigenetic mechanisms	Mouse Human	Ouyang et al. (2013)
Liver	Co-repressor of the retinoic acid-related orphan receptors, RORα and RORγ; negative regulation of circadian clock and metabolic networks	Mouse Human	Takeda and Jetten (2013), Jetten (2009)
Vascular Endothelium	*miR-181α* directly binds to the *3’UTR Prox1* sequence; negative regulation of Prox1 expression	Mouse	Kazenwadel et al. (2010)
Lymphatic Endothelium	Specification of lymphatic endothelial identity by forming heterodimers with COUP-TFII (NR2F2)	Mouse Human	Lee et al. (2009), Yamazaki et al. (2009), Aranguren et al. (2013), Srinivasan et al. (2010)
Hepatocellular Carcinoma	Promotion of metastasis by inducing the expression and protein stability of HIF-1α (hypoxia-inducible factor 1α)	Human	Liu et al. (2013)
Various Human cells	HIF-1α or HIF-2α can directly interact with the hypoxia-response element *(HRE)* at the *Prox1* promoter and induce its expression	Human	Zhou et al. (2013)

*The factors presented here are also involved in neural cell fate decisions.

Prox1 has also been identified as a co-repressor partner for liver receptor homologue-1 (LRH-1/NR5A2), a critical regulator of liver and pancreas development. The overlapping expression patterns and the direct interaction of these two transcription factors led to the identification of novel molecular mechanisms via which Prox1 and LRH-1 co-ordinately regulate the characteristics of hepatocytes (Qin et al., 2004, 2009; Steffensen et al., 2004; Kamiya et al., 2008; Stein et al., 2014). These findings propose important functions of Prox1 and LRH-1 complex during development of the enterohepatic system and in adult physiology of the liver. Most importantly, *LRH-1* mRNA levels have also been detected in the brain of adult mice (Grgurevic et al., 2005; Gofflot et al., 2007). LRH-1 seems to have significant and specific roles in CNS development among other tissues. Recently, we showed that this orphan nuclear receptor is expressed in the developing spinal cord and facilitates the Prox1-mediated inhibition of Notch1 signaling (Kaltezioti et al., 2010; Stergiopoulos and Politis, 2013). It would also be interesting to further examine whether LRH-1 continues to contribute to Prox1 mode of action during adult neurogenesis in the hippocampus.

Other examples of factors that regulate Prox1 expression are the hypoxia-inducible factors 1α and 2α (HIF-1α/HIF-2α). HIF-1α or HIF-2α can directly interact with the hypoxia-response element (HRE) at the *Prox1* promoter and induce Prox1 expression in response to hypoxia (Zhou et al., 2013). In addition, Prox1 promotes hepatocellular carcinoma metastasis by inducing the expression and protein stability of HIF-1α (Liu et al., 2013). In the brain, HIF-1α has been shown to be involved in neurological symptoms of cerebral ischemia. For example, inhibition of HIF-1α and its downstream genes lead to amelioration of the symptoms and neurological deficits in a rat model of focal cerebral ischemia (Chen et al., 2010). HIF-1α elimination is also neuroprotective in neonatal hypoxic-ischemic injury (Chen et al., 2008). Moreover, HIF-1α ameliorates and reduces neuronal apoptosis in a rat model for spinal cord injury (SCI) (Chen et al., 2013). At last, it was recently reported that up-regulation of HIF-1α expression in NSCs or olfactory ensheathing cells (OECs), used in transplantations for the repair of CNS injury, enabled these cells to more efficiently differentiate towards the neuronal lineage (Wang et al., 2014). All these observations could propose potential synergistic roles for HIF-1α/HIF-2α and Prox1 in regulating NSC differentiation during adult neural fate specification and neurological disease progression.

Additionally, in hepatocytes, Prox1 has been shown to interact with LSD1 (lysine-specific demethylase 1) and cause the recruitment of the repressive LSD1/NuRD complex to specific loci, which leads to the co-repression of transcription through epigenetic mechanisms (Ouyang et al., 2013). Regarding its role in nervous system function, LSD1 is involved in the epigenetic control of the initiation of neuronal differentiation program (Ceballos-Chavez et al., 2012; Fuentes et al., 2012; Han et al., 2014). The delineation of the exact role of LSD1/Prox1 complex in the adult brain as well as the identification of potential cell populations that co-express both Prox1 and LSD1 may provide novel insights into the mechanisms involved in adult NSC fate determination.

Finally, Prox1 has also been reported to function as a novel modulator of retinoic acid-related orphan receptors (RORs) α- and γ-mediated transactivation. In particular, Prox1 acts as a co-repressor and negatively influences the ROR-mediated regulation of circadian clock and various metabolic networks/pathways (Takeda and Jetten, 2013). Although the role of RORs in nervous system is still unclear, RORs have been linked with important functions in cerebellar development and circadian rhythm (regulation of clock genes) (Jetten, 2009). Further studies towards the understanding of the exact role of RORs in CNS will provide new insights into a potential tissue-specific connection and interaction with Prox1. These nuclear receptors may contribute to Prox1 mode of action during adult neurogenesis, since single-nucleotide polymorphisms in RORs have been correlated with increased risk of several psychiatric conditions, including bipolar disorder (Le-Niculescu et al., 2009; Table 2).

## Perspectives

Important pieces to our understanding of molecular and cellular mechanisms of Prox1-mediated regulation of adult neurogenesis have been added the last years, supporting the notion that Prox1 represents a central node in the cell fate machinery of adult hippocampus. Recently, Wnt signaling has been shown to directly regulate Prox1 expression in adult hippocampal neurogenesis. Knowledge from other neural areas during development suggests that additional upstream pathways, including Notch signaling, proneural genes (*Neurog2* and *Mash1*) as well as Sox proteins, might be important factors for Prox1 regulation during adult hippocampal neurogenesis (Figure 1). In addition, studies towards factors implicated in Prox1 activity in non-neural tissues, including HIF-1α/HIF-2α, LSD1 and Nuclear receptors such as COUP-TFII, LRH-1 and RORs, suggest that these factors should also be evaluated in adult hippocampal neurogenesis. Therefore, further effort must be invested on identifying novel Prox1 regulators and ultimately connect the variety of inputs that affect Prox1 levels on the different set of hippocampal cells in the adult DG. Additional complication is likely to be achieved by RNA proteins (Staufen), microRNAs (*miR-181α*) and potentially by lncRNAs (Antoniou et al., 2014) that might be proved important players to fine tune Prox1 activity in cell fate decisions of hippocampal cells during adult neurogenesis.

The pleiotropic actions of Prox1 could be plausibly explained by multiple Prox1 targets. Insights from the embryonic brain and other organs suggest that key cell fate regulators, such as Notch, Olig2, p27-Kip1, Cdc25A and HIF-1α/2α, could also represent downstream targets of Prox1 in the adult DG. In summary, we propose that Prox1 might act as a cross-talk point between diverse signaling pathways and cell fate determinants to achieve specific outcomes during adult DG neurogenesis.

## Conflict of interest statement

The authors declare that the research was conducted in the absence of any commercial or financial relationships that could be construed as a potential conflict of interest.
